# Whole-Genome Sequencing of *Clostridium* sp. Strain FP2, Isolated from Spoiled Venison

**DOI:** 10.1128/MRA.00334-20

**Published:** 2020-04-30

**Authors:** Nikola Palevich, Faith P. Palevich, Paul H. Maclean, Ruy Jauregui, Eric Altermann, John Mills, Gale Brightwell

**Affiliations:** a AgResearch Limited, Grasslands Research Centre, Palmerston North, New Zealand; b Riddet Institute, Massey University, Palmerston North, New Zealand; Broad Institute

## Abstract

*Clostridium* sp. strain FP2 was isolated from vacuum-packaged refrigerated spoiled venison in New Zealand. This report describes the generation and annotation of the 5.6-Mb draft genome sequence of *Clostridium* sp. FP2, which will facilitate future functional genomic studies to improve our understanding of premature spoilage of red meats.

## ANNOUNCEMENT

Vacuum packaging and stringent control of storage temperatures allow the export of chilled fresh meat to distant markets (1). Despite these control measures, premature spoilage of vacuum-packed aged red meat by psychrophilic and psychrotrophic *Clostridium* species results in financial losses and reduced consumer confidence. To date, there are limited data on the prevalence and key risk factors that influence *Clostridia*-associated spoilage of chilled meat or blown-pack spoilage (BPS).

This report presents the annotated draft genome sequence of the *Clostridium* sp. FP2 strain, a Gram-positive, spore-forming, and slow-growing psychrotrophic anaerobe. In 2017, FP2 was originally isolated atAgResearch, Ltd., Palmerston North, New Zealand (−40°24'6.84''S, 175°38'38.29''E) from the meat drip of fully distended and vacuum-packaged spoiled venison in which the meat had a sulfur-like odor with no discoloration. FP2 was selected for whole-genome sequencing to examine its role in BPS due to its positive test result for the industrial Clostridium estertheticum-like real-time PCR assay (2). The amplified rRNA restriction analysis (ARDRA) placed strain FP2 within ARDRA group 5 (C. tagluense-like), with >95% similarity to the *C. tagluense* strain A121^T^ (3), isolated from a permafrost core in the Northwest Territories in Canada (4).

FP2 was cultured anaerobically from the meat drip at 10°C in 10-fold suspensions of prereduced peptone, yeast extract, glucose, and starch (PYGS) broth (2). Genomic DNA was extracted using a modified phenol-chloroform procedure as previously described (5, 6). We prepared a TruSeq Nano library from total genomic DNA and sequenced it using the Illumina MiSeq platform (2 × 250-bp paired-end [PE] reads). Trimmomatic v0.39 (http://www.usadellab.org/cms/?page=trimmomatic) was used to filter the raw reads (Q score of >15 in a sliding window of 4 nucleotides [nt]; minimum length, >36 nt), yielding 2,886,708 PE raw sequences, and *de novo* assembly was conducted with A5-miseq v20169825 (7). A total of 111 scaffolds were obtained, with 115× coverage of a total genome size of 5,555,196 bp, with an estimated G+C content of 30.9%. The largest scaffold was 370,460 bp, and the *N*_50_ value was 259,408 bp. All bioinformatics analyses were performed using default parameters unless otherwise specified. The *Clostridium* sp. FP2 genome sequence presented here, with high similarity to *C. tagluense* strain A121^T^ (4), is a valuable resource for future studies investigating the genetic mechanisms of bacterial spoilage in red meats.

### Data availability.

The whole-genome sequencing (WGS) project for *Clostridium* sp. FP2 was deposited in GenBank under accession number JAAMNI000000000. Raw sequence reads were deposited in the Sequence Read Archive (SRA) under accession number SRR11113222. The WGS and SRA records are associated with BioProject number PRJNA574489.
